# Microstructural parameter estimation in vivo using diffusion MRI and structured prior information

**DOI:** 10.1002/mrm.25723

**Published:** 2015-05-20

**Authors:** Jonathan D. Clayden, Zoltan Nagy, Nikolaus Weiskopf, Daniel C. Alexander, Chris A. Clark

**Affiliations:** ^1^ UCL Institute of Child Health, University College LondonLondonUK; ^2^ Wellcome Trust Centre for NeuroimagingUCL Institute of Neurology, University College LondonLondonUK; ^3^ Laboratory for Social and Neural Systems ResearchUniversity of ZurichZurichSwitzerland; ^4^ Centre for Medical Image ComputingUniversity College LondonLondonUK

**Keywords:** microstructure, dual spin‐echo, twice‐refocused spin‐echo, prior, Bayesian, MCMC

## Abstract

**Purpose:**

Diffusion MRI has recently been used with detailed models to probe tissue microstructure. Much of this work has been performed ex vivo with powerful scanner hardware, to gain sensitivity to parameters such as axon radius. By contrast, performing microstructure imaging on clinical scanners is extremely challenging.

**Methods:**

We use an optimized dual spin‐echo diffusion protocol, and a Bayesian fitting approach, to obtain reproducible contrast (histogram overlap of up to 92%) in estimated maps of axon radius index in healthy adults at a modest, widely‐available gradient strength (35 mT m ${}^{-1}$). A key innovation is the use of influential priors.

**Results:**

We demonstrate that our priors can improve precision in axon radius estimates—a 7‐fold reduction in voxelwise coefficient of variation in vivo—without significant bias. Our results may reflect true axon radius differences between white matter regions, but this interpretation should be treated with caution due to the complexity of the tissue relative to our model.

**Conclusions:**

Some sensitivity to relatively large axons (3–15 μm) may be available at clinical field and gradient strengths. Future applications at higher gradient strength will benefit from the favorable eddy current properties of the dual spin‐echo sequence, and greater precision available with suitable priors. Magn Reson Med, 2015. © 2015 The Authors. Magnetic Resonance in Medicine published by Wiley Periodicals, Inc. on behalf of International Society for Magnetic Resonance in Medicine. This is an open access article under the terms of the Creative Commons Attribution License, which permits use, distribution and reproduction in any medium, provided the original work is properly cited. **Magn Reson Med 75:1787–1796, 2016. © 2015 The Authors. Magnetic Resonance in Medicine published by Wiley Periodicals, Inc. on behalf of International Society for Magnetic Resonance.**

## INTRODUCTION

Diffusion‐weighted magnetic resonance imaging uses the random self‐diffusion of water molecules as the basis for an endogenous contrast in biological tissues 1. A greater diffusivity is associated with greater attenuation in the MR signal due to the dispersion of “labeled” molecules during the course of an imaging experiment. Diffusion tensor imaging takes advantage of the orientational dependence of diffusivity in tissue to infer the arrangement of structures such as white matter tracts 2, and varying the time during which molecules are allowed to diffuse allows further properties of tissue architecture to be inferred 3.

A recent trend has been to use diffusion‐weighted magnetic resonance imaging in combination with detailed models of tissue microstructure to try to estimate characteristics which are generally more associated with invasive histology than clinical imaging, such as axon radius. The tissue models are typically built up from simple geometric shapes such as cylinders and spheres, but despite their simplicity they may be able to provide more direct tissue microstructure parameters than can be obtained from traditional diffusion‐weighted magnetic resonance imaging analysis 4, 5, 6. These model parameters may in turn offer greater interpretability and sensitivity as biomarkers. In addition to the pioneering work by Stanisz et al., the “AxCaliber” technique has demonstrated the feasibility of recovering axon radius information from MR images of nervous tissue 7. Subsequently, the “ActiveAx” approach has developed the area toward feasibility in vivo using orientationally invariant protocols, to allow axon radii to be estimated throughout the brain, and optimized pulses sequences, to make acquisition times feasible 4. The strength of the magnetic gradients available has been shown to be a key limiting factor for these applications 8, 9, and the lack of strong gradients at most sites is a major barrier to their widespread uptake.

The best choice of diffusion‐weighted pulse sequence for these applications is the focus of ongoing discussion in the literature. In addition to the original pulsed‐gradient spin‐echo sequence developed by Stejskal and Tanner 10, other diffusion‐weighted sequences have been suggested as candidates for more effective tissue microstructure imaging. For example, oscillating gradient sequences allow very short diffusion times to be achieved, and therefore, may have better sensitivity to small axon radii 11, 12. Multiple wave‐vector protocols can help to distinguish between signals from compartments with different shapes 13, and several authors have proposed that they may provide additional sensitivity, beyond that of pulsed‐gradient spin‐echo, for axon radius estimation 14, 15, 16. One can even use a generalized gradient waveform, enforcing only realistic slew rates and balance to ensure that refocussing and a main echo occur 17, 18.

**Figure 1:**
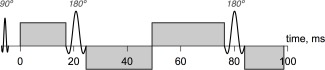
Pulse timing diagram for the standard dual spin‐echo sequence. Only RF pulses and the four diffusion‐weighting gradient pulses are shown for simplicity. Time zero is the earliest time at which a diffusion gradient can first be applied, allowing for the time required for the 90^∘^ excitation RF pulse and other preparatory gradient pulses.

For routine diffusion‐weighted imaging on clinical scanners, the dual spin‐echo sequence (DSE; Refs. 19 and 20) is very popular because it reduces eddy currents at the time of readout, and hence, the image distortions caused by them. It differs from the standard Stejskal–Tanner sequence in that refocussing is applied twice, with four diffusion‐sensitizing gradients appearing around the refocussing pulses (see Fig. 1). It may offer additional benefits for microstructural imaging in terms of sensitivity, due to its allowance for gradient pulses placed next to each other, which produces relatively short effective diffusion times. (With pulsed‐gradient spin‐echo on clinical hardware, by contrast, a lower bound is imposed by the time required for the radiofrequency refocussing pulse.) The tradeoff is that a longer echo time is needed for DSE, reducing the available signal. We have previously derived an expression for the signal expected from this sequence within impermeable cylinders 21, using the Gaussian phase distribution approximation 22. We further adapted the experimental design optimization from Ref. 23 for the DSE sequence using this signal model, and demonstrated the sequence's potential advantages for estimating small axon radii, in particular, using simulations 21.

In this study, our aim is to investigate whether meaningful axon radius information can be obtained in practice using standard scanner hardware and a widely available pulse sequence. Specifically, we apply an optimized DSE sequence on a standard 3 T clinical scanner, using a maximum gradient strength of 35 mT m ${}^{-1}$. We consider the corpus callosum in the human brain, where the distribution of axon radii is well characterized by postmortem histology 24. This structure has been well studied in the broader imaging literature, as damage to it has been shown to have a role in a number of disease processes. However, microstructural measurements at the modest gradient strength applied here are sensitive to radii only at the very upper limit of those observed in human callosal tissue. To improve precision we use a standard model together with a new parameter estimation algorithm that incorporates prior information about plausible radii. We use simulations to demonstrate that the algorithm provides contrast between large and small radii, without substantial bias, under idealized conditions. In brain data acquired from adult volunteers, axon radius index maps consistently indicate the presence of large axons in the same regions suggested by histology, in particular in the anterior mid‐body of the corpus callosum.

## METHODS

We begin by outlining our signal model, and the processes we applied for optimizing the DSE sequence, acquiring data and fitting tissue model parameters.

### Signal Model

The diffusion‐weighted signal in white matter, *S*, is modeled as a weighted sum of signal contributions from three compartments: an isotropic compartment representing cerebrospinal fluid contamination, a restricted “intracellular” compartment, and a hindered “extracellular” compartment (cf. Refs. 4 and 25). We denote the isotropic volume fraction with *f*_i_ and the restricted volume fraction with *f*_r_, subject to $0\leq f_{i}+f_{r}\leq 1$. Then,

(1) $$\frac{S(\Theta ,f_{i},f_{r})}{S_{0}}=f_{i}S_{i}(\Theta )+f_{r}S_{r}(\Theta )+(1-f_{i}-f_{r})\;S_{h}(\Theta )\;,$$

where *S*_i_, *S*_r_, and *S*_h_ are the signals from the isotropic, restricted, and hindered compartments respectively, *S*_0_ is the signal without diffusion weighting, and Θ is a set of additional parameters. The isotropic signal component is a simple function of the standard diffusion *b*‐value and the diffusivity, *D*_i_, of free water, viz. $S_{i}=\exp(-bD_{i})$.

The white matter tissue of interest is modeled as a coherent bundle of parallel, impermeable, hollow cylinders of fixed radius, *R*. The extracellular, hindered compartment is assumed to be homogeneous, and diffusion is assumed to follow a cylindrically symmetric 3D Gaussian distribution, viz.

(2) $$S_{h}(\Theta )=\exp\left(-b\left(\cos^{2}\alpha \;(D_{∥}-D_{⊥})+D_{⊥}\right)\right)\;,$$

where *α* is the angle between the gradient direction and the orientation of the white matter bundle, $D_{∥}$ is the diffusivity parallel to the cylinders, and $D_{⊥}$ is the diffusivity perpendicular to them.

Following Neuman and van Gelderen et al. 26, 27, we have previously derived an expression for the restricted diffusion signal within cylinders for the DSE sequence 21, using the Gaussian phase distribution approximation 22. That result is used here for *S*_r_, unmodified. It depends on the cylinder radius, *R*; the intracellular diffusivity, which we take as equal to $D_{∥}$; and the orientation of the cylinder, which we parameterize using the spherical coordinate angles, *θ* and ϕ.

The full parameter set is therefore, $\Phi =\{S_{0},f_{i},f_{r},D_{i},D_{∥},D_{⊥},R,\theta ,\phi \}$.

We note that this model does not take into account differences in *T*_1_ or *T*_2_ relaxation times in the different compartments, whereas in practice such differences will exist. However, the isotropic volume fraction is expected to be very small in most voxels, and so the influence of this limitation on parameter estimates will be minimal. Moreover, *f*_i_ and *D*_i_ are treated as nuisance parameters of little interest in this study.

### Sequence Optimization

The experiment design optimization framework developed by Alexander 23 was used to optimize a DSE imaging protocol for estimating the tissue parameters of interest. This framework aims to identify combinations of pulse arrangements, within the constraints imposed by the sequence and the performance of the scanner, which will maximize the expected precision of the tissue parameters, using the formalism of the Cramér–Rao lower bound.

The generative parameters used for the optimization were: $S_{0}=1$, *f*_i_ = 0, $f_{r}=0.7,\;D_{i}=3\times 10^{-9}$ m ${}^{2}$ s ${}^{-1},\;D_{∥}=1.7\times 10^{-9}$ m ${}^{2}$ s ${}^{-1}$, and $D_{⊥}=1.2\times 10^{-9}$ m ${}^{2}$ s ${}^{-1}$, following Ref. 21. Generative radii were R∈{5,10,20} μm, as in Ref. 4, with the optimization seeking a combination of pulse arrangements that jointly minimize average expected variance in the model parameters across these three values (although large relative to the expected radii in most tissue, they represent the domain where we expect some sensitivity at low gradient strength). No assumptions were made about the orientations of the axon bundles within the voxel, and gradient directions for each arrangement were therefore uniformly spread over the sphere. For the purposes of estimating the noise properties of the protocol, the spin–spin relaxation constant, *T*_2_, was taken to be 0.07 s. The number of separate diffusion‐weighted pulse arrangements was fixed to four, and the number of gradient directions per arrangement was fixed to 90. A *b* = 0 arrangement was also included.

Eddy currents with a time constant of $0.7/\tilde{T}$ were nulled, with $\tilde{T}$ the maximal sum of all diffusion‐encoding gradient pulse lengths across the five arrangements, as proposed by Heid 28. Off‐design eddy current effects are also reduced by this process, which removes one degree of freedom from the optimization.

**Figure 2:**
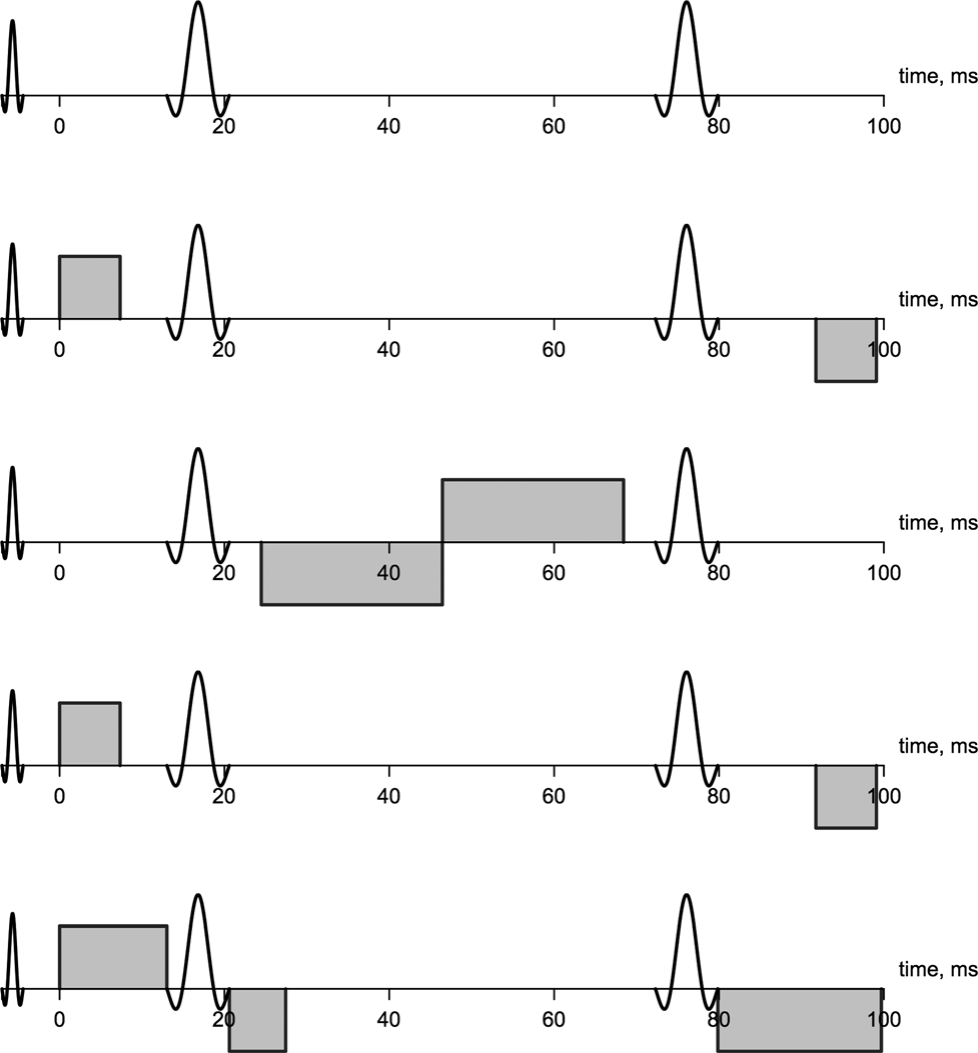
Optimized series of five pulse arrangements, with *b*‐values of 0, 422, 620, 422, and 2378 s mm ${}^{-2}$. Note that all four standard pulses do not actually exist in any one arrangement, but all are properly balanced. The second and fourth arrangements are in fact identical.

The optimized pulse arrangements are shown in Figure 2, and precise gradient timings are given in Table 1. The diffusion *b*‐values corresponding to the five pulse arrangements were 0, 422, 620, 422, and 2378 s mm ${}^{-2}$. Gradient amplitude in each case, except where *b* = 0, was the maximum allowed, at 35 mT m ${}^{-1}$. The echo time was included in the optimization but not allowed to vary across arrangements; its final value was 118.54 ms. In general, the DSE sequence has no specific diffusion time associated with it, but for the simple arrangements in Figure 2, we can compute an effective diffusion time in a similar way to the pulsed‐gradient spin‐echo sequence. On this basis, arrangements 2, 4, and 5 have long diffusion times (80–92 ms), while arrangement 3 has a short diffusion time (22 ms).

**Table 1:** Pulse Timings, in Milliseconds, as Implemented in the Final Protocol Shown in Figure 2.

	Pulse arrangement				
	1	2	3	4	5
Onset 1		0.00		0.00	0.00
Length 1		7.34		7.34	13.01
Onset 2			24.47		20.60
Length 2			21.97		6.83
Onset 3			46.45		
Length 3			21.97		
Onset 4		91.76		91.76	79.86
Length 4		7.34		7.34	19.84
*b*‐value, s mm ${}^{-2}$	0	422	620	422	2378

Time zero is the earliest time at which a diffusion gradient can first be applied, allowing for the time required for the 90^∘^ excitation RF pulse and other preparatory gradient pulses. Missing values correspond to omitted pulses. Echo time is 118.54 ms in all five pulse arrangements. The time required for a 90^∘^ RF pulse was 2.56 ms, and 7.56 ms was required for a 180^∘^ pulse and its associated crusher gradients

### Synthetic Data

Synthetic data were obtained using Monte Carlo simulation, as implemented in Camino 29, 30. The simulation tracks the phase of 10,000 spins over 1000 time steps during the optimized pulse arrangement, to calculate the final signal. The simulated tissue geometry consisted of hexagonally packed impermeable cylinders of fixed radius, with a universal diffusivity of $1.7\times 10^{-9}$ m ${}^{2}$ s ${}^{-1}$. Run time was approximately 5 min on a standard iMac desktop computer.

The simulation was carried out for axon radii of 1, 3, 5, 10, 15 and 20 μm, with the center‐to‐center cylinder separation fixed at 2.3 times the radius, to maintain an intracellular volume fraction of 0.69 in all cases. Ten different axon orientations were used for each radius, equally distributed on the sphere. Rician noise was added to the simulated signals, based on an SNR of 19 at *b* = 0 with the optimized echo time, matching the estimated noise characteristics of the scanner.

In addition, synthetic substrates containing a range of axon radii, distributed according to gamma distributions with means of 3, 5, and 8 μm and variances of 2.5 μm were also generated and fitted using our model. In this case, cylinders were placed at random, but intracellular volume fractions were always between 0.65 and 0.70. This experiment was intended to test the influence of a particular type of mismatch between the data and the model.

Finally, to investigate axon radius estimation at different gradient strengths, the protocol was reoptimized using a maximum gradient strength of 300 mT m ${}^{-1}$, in line with the top end of what is currently available on human scanners 31. In this case, synthetic data were generated using a single axon radius of 3 μm.

### Data Acquisition and Preprocessing

Although the gradient strength capabilities of the scanner, as well as the time needed for RF pulses, preparation pulses, and readout are taken into account by the optimization, it does not incorporate slew rate information. It was therefore necessary to remove some very short pulses from the optimized arrangements to make them realizable in practice. The other pulse lengths were adjusted as necessary to restore balance, and the pulse diagrams in Figure 2 incorporate these edits.

Three individuals—a 23 year‐old female, a 32 year‐old male, and a 31 year‐old female—were each scanned on two separate occasions on a Siemens Trio 3 T clinical scanner, using a body transmit coil and vendor‐supplied 32 channel receive‐only head coil, as well as a standard gradient coil set ( $G_{\text{max}}=35$ mT m ${}^{-1}$). The optimized protocol was applied, consisting of ten *b* = 0 images followed by sets of 90 identical diffusion sensitizing directions for each of pulse arrangements 2–5, shown in Figure 2. Data were acquired from a series of contiguous axial slices covering the corpus callosum, at 2.3 mm isotropic resolution. Scan time was approximately 1 h, but cardiac pulse triggering was used, and so the exact time depended on each subject's heart rate. Three slices were imaged per heartbeat.

DICOM files were converted to NIfTI format using the TractoR software package 32. The first *b* = 0 volume was treated as a dummy and removed from each data set. Due to the favorable eddy current properties of the DSE sequence, and a high degree of cooperation from scan subjects, the image volumes were observed to be well aligned. Therefore no coregistration was performed, to avoid introducing spurious motion and blurring in the data. Diffusion tensors were fitted to the data using ordinary least squares and a mask of the corpus callosum was drawn by hand by a single observer on one midsagittal slice in each data set, using the fractional anisotropy map as a guide. Parameter fits were then performed within this mask.

### Parameter Fitting

Equation (1) gives an expression for the expected signal from our simple tissue substrate given known tissue parameters. Given a set of measured signal values, we need to solve the inverse problem, finding the set of parameters, $\hat{\Phi }$, which best explain the measurements. As we have some expectations regarding the regime of values for many of the parameters, we would also like to make use of that information. We therefore take a Bayesian approach, estimating a posterior distribution over the parameters using Markov chain Monte Carlo (MCMC).

We assume that measurements of the signal, $x_{\left(k\right)}$, are drawn from a Rician distribution around the modelled value from Eq. (1), $S_{\left(k\right)}$, viz.

(3) $$P(x_{\left(k\right)}|\Phi ,\sigma )=\frac{x_{\left(k\right)}}{\sigma^{2}}\exp\left(\frac{-(x_{\left(k\right)}^{\;2}+S_{\left(k\right)}^{\;2})}{2\sigma^{2}}\right)I_{0}\left(\frac{x_{\left(k\right)}S_{\left(k\right)}}{\sigma^{2}}\right)\;,$$

where *σ* controls the noise level. $I_{0}(\cdot )$ is the modified Bessel function of the first kind, order zero. If the noise associated with each measurement can be considered to be independent and identically distributed, then we can write down the joint distribution of the full set of measurements, $X=(x_{\left(k\right)})$, as

(4) $$P(X|\Phi ,\sigma )=\prod_{k}P(x_{\left(k\right)}|\Phi ,\sigma )\;.$$

Bayes’ rule then allows us to calculate a posterior distribution over the parameters as

(5) $$P(\Phi ,\sigma |X)=\frac{P(X|\Phi ,\sigma )P(\Phi )P(\sigma )}{\int P(X|\Phi ,\sigma )P(\Phi )P(\sigma )}\;,$$

where P(Φ) represents the prior information available regarding Φ, and P(σ) likewise for *σ*. (We assume prior independence of Φ and *σ*.)

At this point, we could simply choose “uninformative” priors for each parameter, ensuring only that physical requirements such as positivity are met by the estimates. However, estimating our tissue model parameters using data from a scanner with clinical gradient strengths is expected to yield relatively low precision, and by choosing more influential priors we regularize the problem. To this end, the following informative priors were used across all model fits:

(6) $$\begin{array}{c} f_{i}∼\text{Beta}(1.2,1.2)\;,\\ \frac{f_{r}}{1-f_{i}}∼\text{Beta}(5,5)\;,\\ D_{∥}∼\text{Log}‐N(-20.69,1)\;,\\ D_{⊥}∼\text{Log}‐N(-21.04,1)\;,\\ R∼\text{Gamma}(3.562,1.404\times 10^{-6})\;. \end{array}$$

These values are based on SI length and time units, that is, meters and seconds. We use $f_{r}/(1-f_{i})$ rather than *f*_r_ directly to give the parameter fixed bounds of 0 to 1. The two volume fractions have nonuniform distributions to regularize the posterior away from the extremes. The log‐normal distribution has been used before as a prior for diffusivity parameters, for example by Andersson 33, and the means of the distributions given above correspond to the values used for optimization, that is, $1.7\times 10^{-9}$ m ${}^{2}$ s ${}^{-1}$ for $D_{∥}$ and $1.2\times 10^{-9}$ m ${}^{2}$ s ${}^{-1}$ for $D_{⊥}$. The gamma distribution—parameterized here using shape and scale parameters—has likewise been used to represent distributions over axon radii (e.g. Ref. 7), and our values are chosen to give the distribution a mean of 5 μm, approximately 4–5 times the mean observed across the corpus callosum in histological studies such as Ref. 24, in line with previous findings of overestimation of the histological mean axon radius by the estimated axon radius index from diffusion‐weighted magnetic resonance imaging 4, 8.

Despite our use of informative prior distributions for the parameters described above, we do not explicitly impose any distributional assumptions on the posterior distributions. These are represented by the empirical distributions of the values sampled from the MCMC algorithm.

A series of samples were drawn from the posterior distribution, Eq. (5), using a blocked Metropolis–Hastings algorithm, with a tuned multivariate Gaussian proposal distribution for each block. The parameter blocks in this case were $\left(S_{0}\right)$, (*f*_i_, *f*_r_), $\left(D_{∥},D_{⊥},R),\;(\theta ,\phi \right)$, and (σ). The isotropic diffusivity, *D*_i_, was fixed at $3.0\times 10^{-9}$ m ${}^{2}$ s ${}^{-1}$ to represent free water, and not sampled. For the synthetic data only, *f*_i_ was also fixed at zero.

A least‐squares tensor fit was first performed for each voxel, yielding principal diffusivities $\lambda_{1}\geq \lambda_{2}\geq \lambda_{3}$. MCMC chains were then initialized according to

(7) $${\tilde{f}}_{i}=\frac{\lambda_{3}^{2}}{\lambda_{1}\lambda_{2}}\;\;{\tilde{f}}_{r}=0.7\;(1-{\tilde{f}}_{i})\;\;{\tilde{D}}_{∥}=\lambda_{1}\;\;{\tilde{D}}_{⊥}=\frac{\lambda_{2}+\lambda_{3}}{2}\;\;\tilde{R}=5\;\mu m\;.$$

An iterative process was then used to tune the covariance matrix for each proposal distribution, bringing each block acceptance rate close to the theoretical optimum of around 23% so that the parameter space would be explored efficiently (cf. Ref. 34). Afterward, the chain was run for a burn‐in period of 50,000 steps, followed by a sampling phase gathering 50 samples of each parameter, with each sample separated by 100 steps. In practice, samples were actually of the logit or logarithm of parameters with bounds, to ensure that the sample space was infinite.

The algorithm was implemented on the TractoR platform in R and C++, using the “Rcpp” and “RcppArmadillo” libraries for the R statistical language and environment 32, 35, 36, 37.

The consistency of the MCMC over multiple chains was assessed, and samples were checked visually in several voxels for evidence of good mixing and convergence to the stationary posterior distribution.

### Histogram Similarity

The histogram intersection measure introduced by Swain & Ballard 38 was used to quantify the similarity of axon radius distributions across scans. This normalized measure is based on the observed probability density in each bin, *j*, of the two histograms, *h*_1_ and *h*_2_. Specifically,

(8) $$H=\frac{\sum_{j}\min\left\{h_{1}^{j},h_{2}^{j}\right\}}{\sum_{j}h_{1}^{j}}\;.$$

Intuitively, *H* represents the proportion of the histogram which is common to both data sets, and it therefore has a range of 0 to 1. As the denominator will evaluate to the inverse of the bin width, this measure is symmetric as long as the bins are the same in each histogram.

## RESULTS

**Figure 3:**
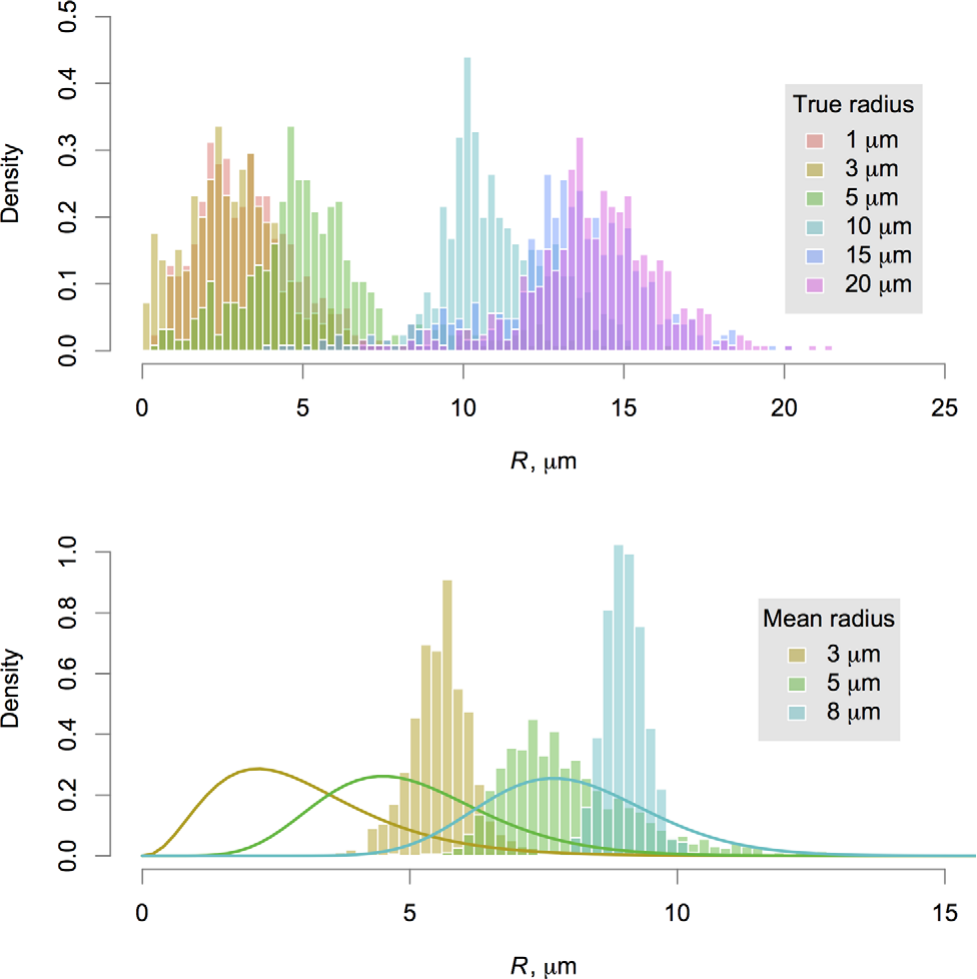
Histograms of sampled posterior axon radius values from our synthetic data, using a single fixed cylinder radius (top) or a gamma distribution of radii (bottom). For the former, samples cover ten axon orientations at each radius. For the latter, the generative distributions (shown) have means of 3, 5, and 8 μm, and variances of 2.5 μm.

**Figure 4:**
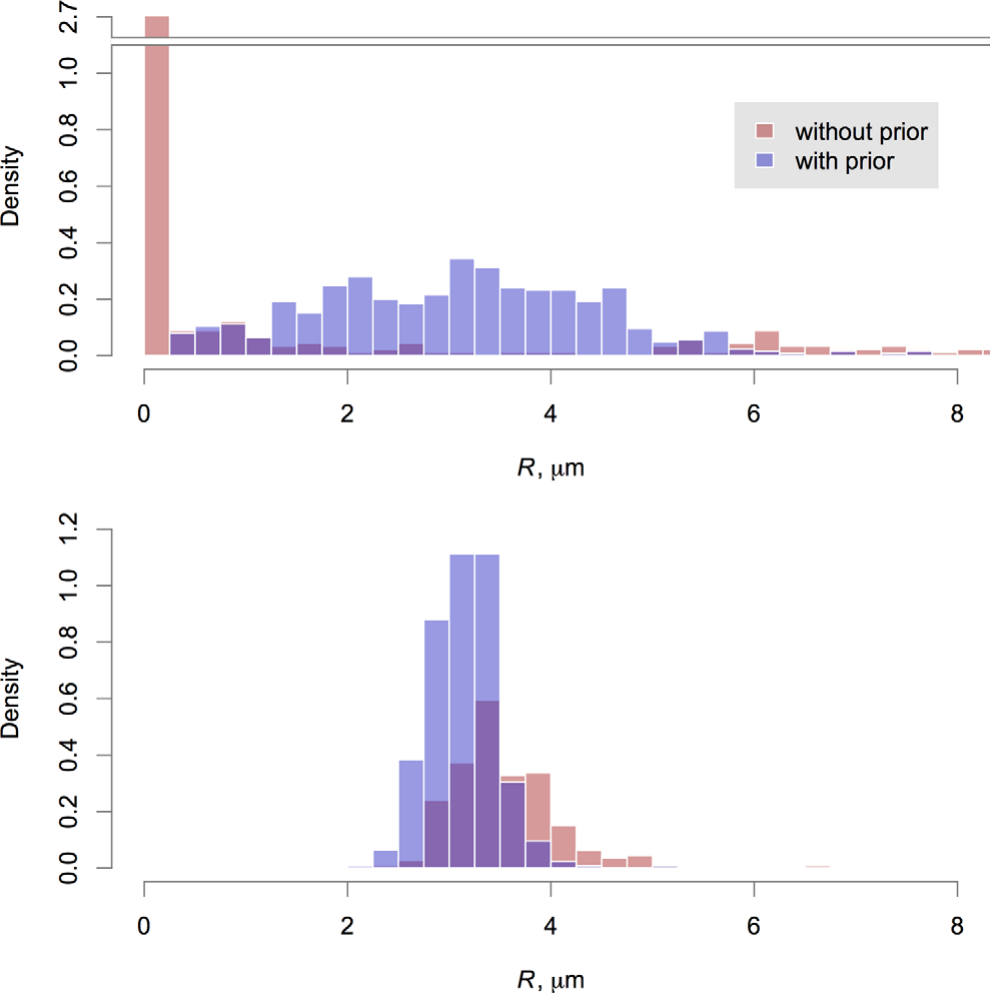
Histograms of sampled posterior axon radius from synthetic data, based on scan protocols optimized for maximum gradient strengths of 35 mT m ${}^{-1}$ (top) and 300 mT m ${}^{-1}$ (bottom). In each case, samples are shown from fits performed both with (blue) and without (red) applying the gamma prior distribution on *R*. The true generative axon radius was 3 μm, and samples cover ten axon orientations. Only samples below 8 μm are shown. Note that the *y*‐axis in the upper plot is broken to accommodate a very tall bar.

Figure 3 shows histograms of sampled axon radii from the synthetic data. At the low end of the scale, precision is too poor to distinguish radii of 1 and 3 μm (*H* = 0.84, using bins of width 0.25 μm); and at the high end, the influence of the prior forces underestimation of very large radii of above 15 μm. However, radii of 3, 5, 10, and 15 μm are clearly discriminable, with sample medians closely approximating the true, generative radius in each of these cases (*H* = 0.41 between 3 and 5 μm; *H* = 0.12 between 5 and 10 μm; *H* = 0.44 between 10 and 15 μm). When the synthetic substrate contains a gamma distribution of radii, as shown in the bottom part of the figure, there is a bias in the posterior distributions, but it remains possible to distinguish larger radii from smaller ones. Figure 4 demonstrates that much greater precision is available at 3 μm when gradient strengths increase to 300 mT m ${}^{-1}$, but it improves still further when prior information is used.

**Figure 5:**
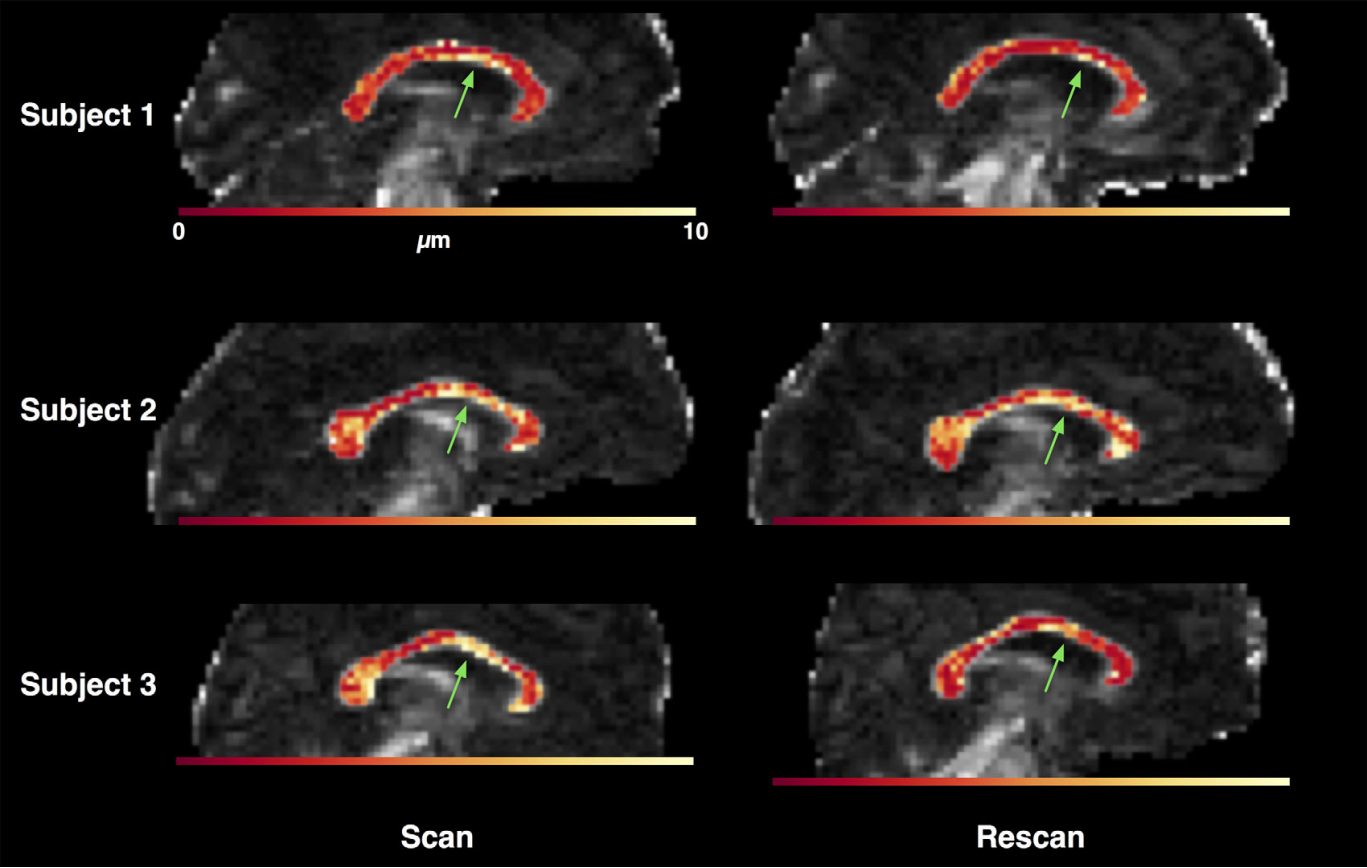
Maps of axon radius index, estimated as the median of the posterior sampled values in each voxel. An area of relatively large axons is consistently observed in the anterior mid‐body of the corpus callosum (green arrows). Subjects are in rows, and scans in columns. The underlying greyscale map is fractional anisotropy, and the color scale is the same in all subfigures.

Maps of axon radius index, the median sampled value of *R* in each voxel, are shown for in vivo data in Figure 5. A fair degree of consistency was observed, both between the first and second scans for each subject, and between subjects. The maximal radius index was generally observed in the anterior mid‐body region and, in subjects 2 and 3, the splenium. Histograms across the whole corpus callosum showed substantial overlap between each pair of scans (*H* = 0.92 for subject 1, 0.89 for subject 2, and 0.76 for subject 3, using bins of width 0.5 μm), and a similar level of overlap between subjects (*H* between 0.78 and 0.91).

**Figure 6:**
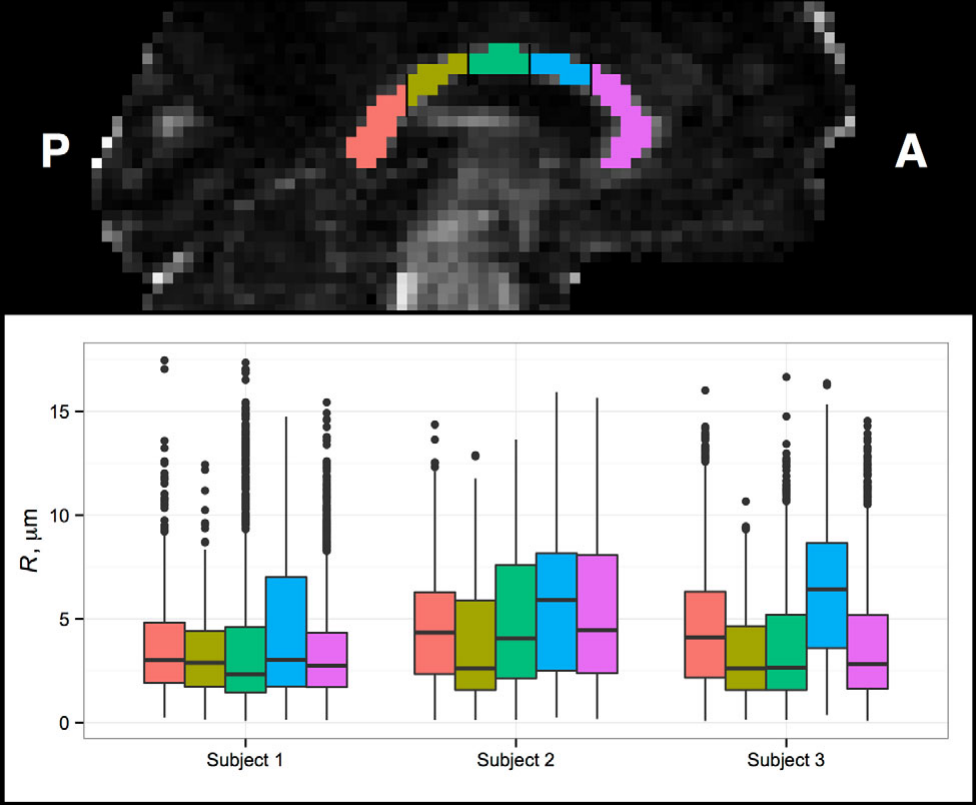
Boxplots of sampled posterior axon radius index in five subregions of the corpus callosum, across both scans, in each subject. In each case median axon radius index was highest in the anterior mid‐body (blue). Filled circles represent outliers more than 1.5 interquartile ranges above the third quartile.

To further examine variation along the corpus callosum, we divided each subject's segmented region of interest into five subregions, separated by equally spaced coronal planes, similar to Aboitiz et al. 24. Boxplots of sampled axon radius index in each subregion for each subject are shown in Figure 6. The anterior mid‐body subregion (shown in blue) has the highest radii on average in every subject, although the degree of variation across subregions is small in subject 1. The scan–rescan relative absolute difference, viz. |a−b|/(a+b), in the median axon radius on a subregion‐by‐subregion basis varied from 11% (posterior mid‐body) to 24% (anterior mid‐body).

The model was a good fit to the acquired data, in terms of the proportion of total signal variance explained. Voxelwise coefficients of determination (*R* ^2^) averaged between 0.84 and 0.93 for each of the six scans.

**Figure 7:**
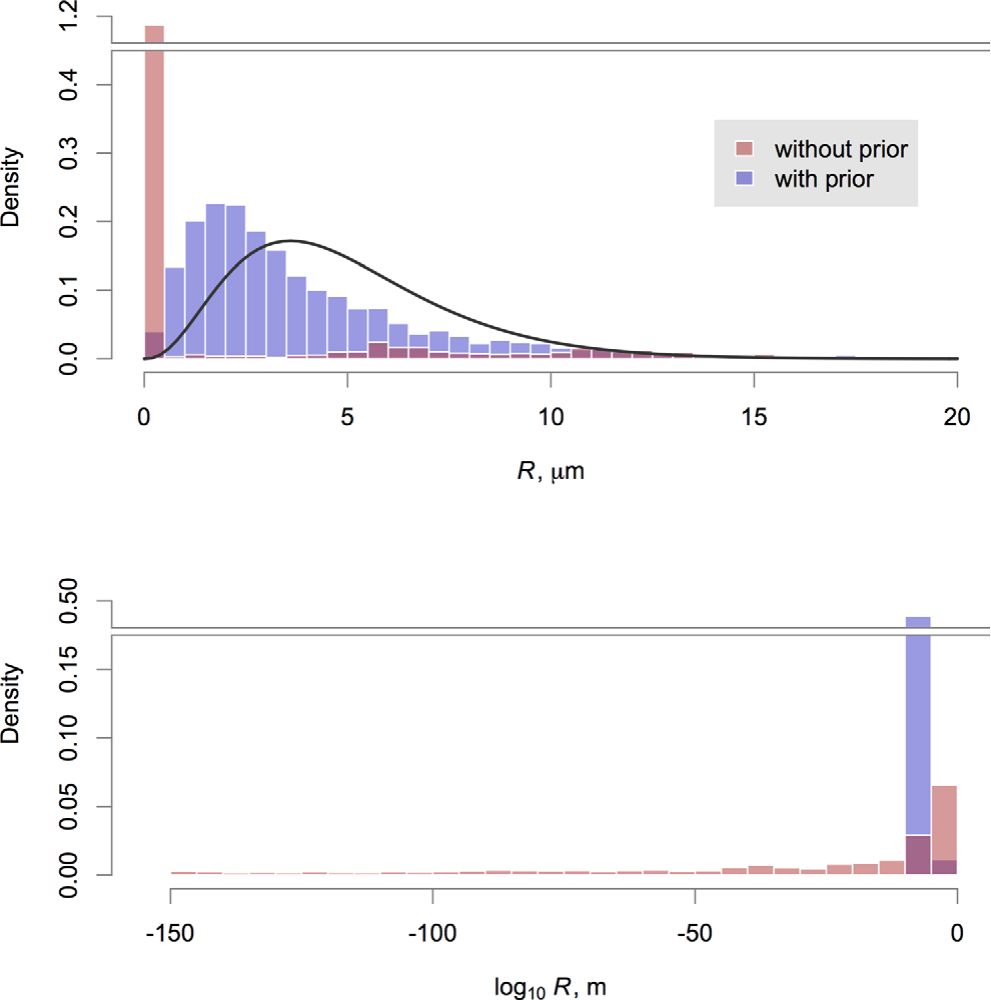
Histograms of sampled posterior axon radius index values across the entire corpus callosum, in the first scan of the first subject. Samples are shown from fits performed both with (blue) and without (red) applying the gamma prior distribution (black curve). The upper figure uses a standard *x*‐axis and shows just those samples below 20 μm, while the bottom figure shows all samples on a logarithmic *x*‐axis. Note that both *y*‐axes are broken to accommodate very tall bars.

**Figure 8:**
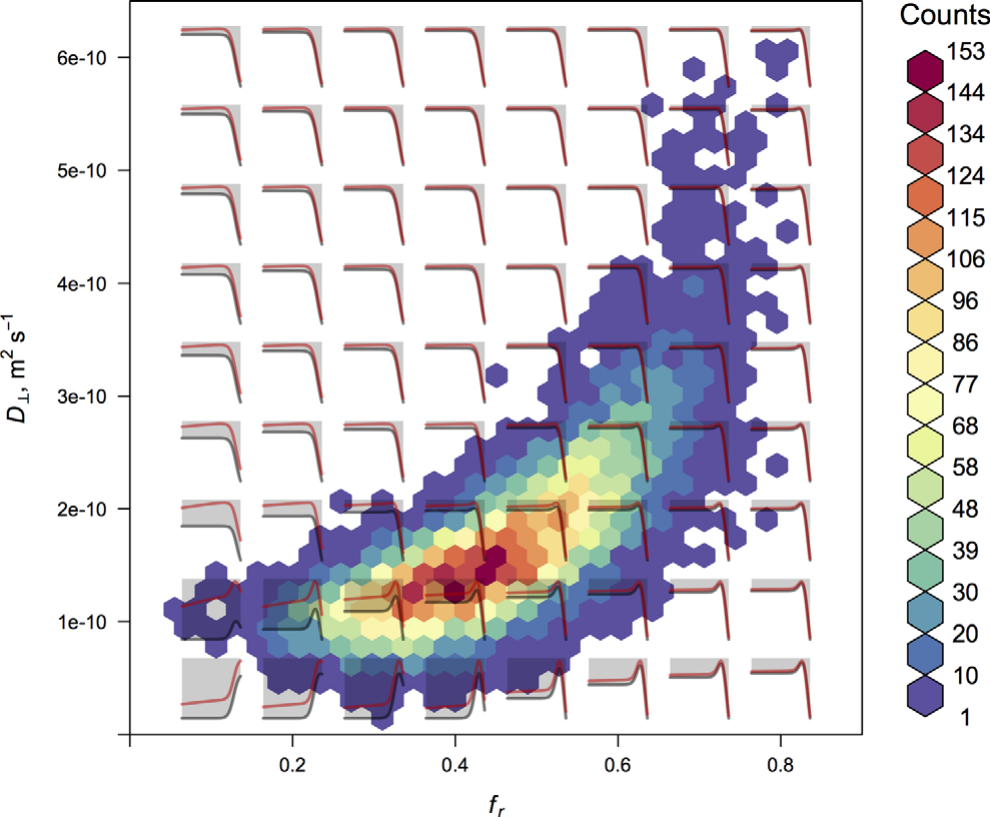
Joint histogram of the marginal posterior distribution over *f*_r_ and $D_{⊥}$ in one voxel in the center of the splenium in subject 2, scan 1, based on 10,000 samples. Overlaid are plots of log likelihood (black) and log posterior (red) as functions of log axon radius, over the range 0.02 to 20 μm, conditioned on the values of *f*_r_ and $D_{⊥}$ corresponding the relevant location in the histogram. These overlaid plots are not to scale with one another, and are instead intended to show the shapes of the relevant functions.

To illustrate the influence of the priors used in our fit, Eq. (6), we show in Figure 7 all of the sampled values for the axon radius parameter for one scan, both with and without the prior in use (priors on all other parameters were retained in the latter case). We can observe that the prior is strongly influential, as there is an extremely wide range of sampled radii in its absence. The influence of the data, encapsulated in the Bayesian likelihood term, may therefore be considered to be relatively weak, which is to be expected at clinical gradient strengths. However, Figure 8 shows that in regions of substantial probability mass with respect to *f*_r_ and $D_{⊥}$, the likelihood does show a substantial peak in the micron range of axon radius. It also illustrates that the prior influences the chains away from the very small radii observed without it, in Figure 7.

**Figure 9:**
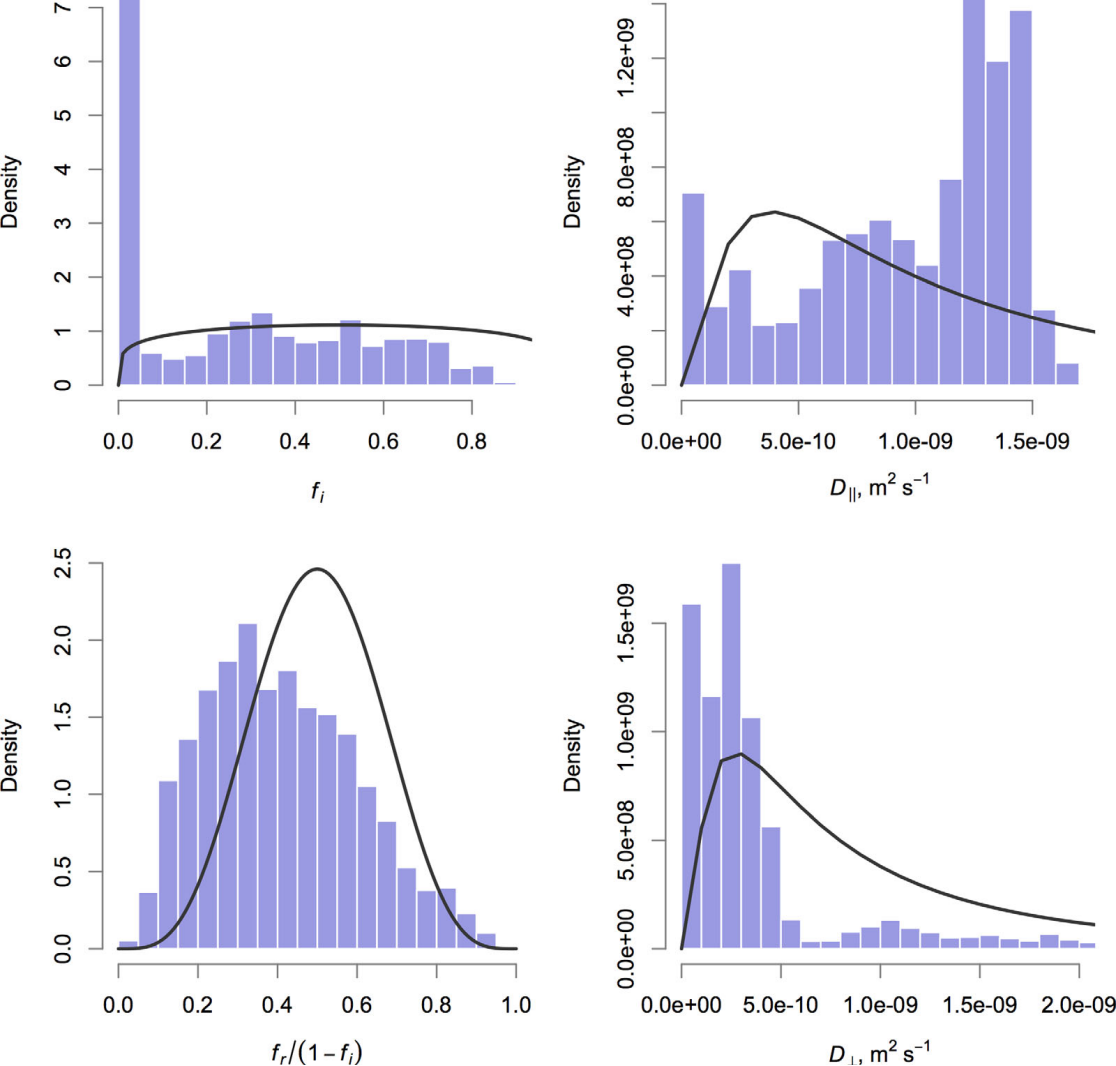
Histograms of sampled posterior values for each parameter with a prior distribution, except axon radius index which is shown in Figure 7. The prior itself is shown as a black curve. The posterior distributions differ substantially from the priors in each case, demonstrating the influence of the data.

Marginal posterior distributions for all other parameters with priors are shown in Figure 9, and in each case there are substantial differences between the prior and posterior distributions, indicating the influence of the data.

Axon radius index maps obtained without use of the prior on *R* are shown in Supporting Information Figure S1. Although some of the same areas of higher radius are still just about visible, the maps appear more noisy and demonstrate less consistency and smoothness. Reproducibility is also substantially lower in this case: in subject 1, for example, *H* decreases from 0.92 to 0.69. Voxelwise coefficients of variation averaged 311% without the prior, across all subjects, compared with 42% with the prior.

## DISCUSSION

Our key contribution in this article has been to demonstrate that structured prior information can improve precision in microstructural parameter estimates. Using this platform, we have explored the application of the ActiveAx approach to in vivo axon radius imaging using the popular DSE pulse sequence on a clinical scanner. Such a standard MRI system is suboptimal for this kind of work, but commonly available, and our results inform the feasibility of “histological” imaging on typical hardware and with a standard pulse sequence, albeit one which has been optimized for the task. We used Bayesian MCMC simulation to estimate the tissue model parameters of interest, formally accounting for both prior expectations and the information available in the data.

Given the challenges associated with microstructure imaging on this platform, we found that it was necessary to regularize the problem by using informative prior distributions for several parameters, and particularly for the axon radius itself. We showed that this information improves precision at intermediate values of axon radius index (Fig. 7), without introducing significant bias when the assumption of a single radius holds (Fig. 3). Synthetic data based on a substrate containing a distribution of radii did lead to bias, most likely because of the greater signal contribution from within wider cylinders. Nevertheless, it was still possible to tell distributions with different means apart a posteriori, with their ordering preserved.

Because of the strong influence of the priors, it cannot be said that we are truly estimating axon radius, as the range of acceptable values is effectively imposed by the prior. Moreover, we cannot demonstrate any improvement in accuracy, as ground‐truth data are not available in vivo. Nevertheless, it is reasonable to interpret the relative differences from voxel to voxel as corresponding to meaningful variation across, in this case, the corpus callosum—and likewise, results are comparable across subjects since the priors are the same in each case. Scan–rescan consistency was seen to be reasonable, both visually and in terms of a quantitative histogram intersection measure. Subject 3 was the least consistent, possibly due to small within‐volume movements during scanning or other external factors.

Our experiment with synthetic data suggested that our fitting process should provide sensitivity to axon radii of around 3–15 μm (Fig. 3). The contrast in the in vivo results may therefore reflect the detection of relatively large axon radii in certain parts of the corpus callosum—although our fits from substrates containing gamma distributions of radii suggest that while qualitatively meaningful, our axon radius index values are likely to be overestimates. Nevertheless, while Alexander et al. 4 and Dyrby et al. 8 have suggested that only very weak sensitivity to axon radius index is available at clinical gradient strengths, we have shown that using suitable priors can ameliorate the situation.

This advantage applies even at the upper end of what is currently achievable on human systems (cf. Fig. 4, where precision is shown to be higher with the prior than without it). Moreover, as eddy current distortions are worse at higher gradient strengths, the favorable eddy current properties of the DSE sequence will also be particularly useful in this regime. The combination of the DSE sequence and informed fitting process may therefore be a powerful one for future microstructural imaging studies on the next generation of scanner hardware—although there are other practical challenges to overcome, such as signal loss due to the greater influence of concomitant fields 39. It will also be important for future work to compare DSE against oscillating gradient sequences and other alternatives, in combination with suitable priors, to fully evaluate their relative merits.

Although the trends we observe suggest that our technique may act as a detector for voxels containing larger axons, the results must be interpreted with caution because of the simplicity of the tissue model used. The three compartment model described by Eq. (1) ignores a great deal of the complexity in real tissue, even in relatively coherent and homogeneous areas of white matter such as the corpus callosum. There has been recent work adding characteristics such as membrane permeability and fiber dispersion and crossing to similar models 40, 41, 42, and distributions of axon radii were included in the AxCaliber model 7. Other factors which may need to be taken into account include the possibility of different *T*_2_ relaxation constants in different tissue compartments (cf. Refs. 43 and 44). Hence, further investigation using more complex models will be required to clarify the source of the contrast observed in our axon radius index maps (Fig. 5). There are also some limitations to our sequence parameterization, such as the assumption of rectangular—rather than trapezoidal—pulses, which were not addressed so as to keep the theoretical signal model analytically tractable. The latter may, however, have only a small influence 45. Finally, we optimized the sequence for axon radii larger than those actually expected in tissue, to make the optimization stable—although it is unlikely that sensitivity to smaller radii could be improved substantially using the same scanner hardware.

In conclusion, we have found that some sensitivity to relatively large axons may be available using the standard DSE pulse sequence at clinical field and gradient strengths, if the scan is well set up and parameter estimation is performed carefully, with the use of suitable prior information. We have demonstrated a consistent trend in axon radius parameter maps which is broadly in keeping with known tissue characteristics, although caution is required in the interpretation of this finding. It may be that contrast arises from large axons of radius around 3 μm and above, although it may also arise from variations in microscopic or macroscopic fiber dispersion (cf. Ref. 4646), axonal undulation 47, or from differences in other tissue properties such as membrane permeability. In vivo axon radius imaging on clinical scanners should therefore be treated cautiously at present, but as stronger gradients become available on these scanners—a tendency which is beginning to become reality—results will improve, and inferring tissue characteristics should become more practical.

## Supporting information


**Figure S1**: Maps of axon radius index, estimated in the absence of the prior on *R*. Compared to Fig. 5, the maps are noisier and show less scan–rescan consistency.
