# Supplementary material for: Microstructural parameter estimation in vivo using diffusion MRI and structured prior information
Source: Magn Reson Med. 2015 May 20;75(4):1787–96. doi: 10.1002/mrm.25723 (PMC4791093; doi:10.1002/mrm.25723)

Supporting Figure S1: Maps of axon radius index, estimated in the absence of the prior on R. Compared to Fig. 5, the maps are noisier and show less scan–rescan consistency.

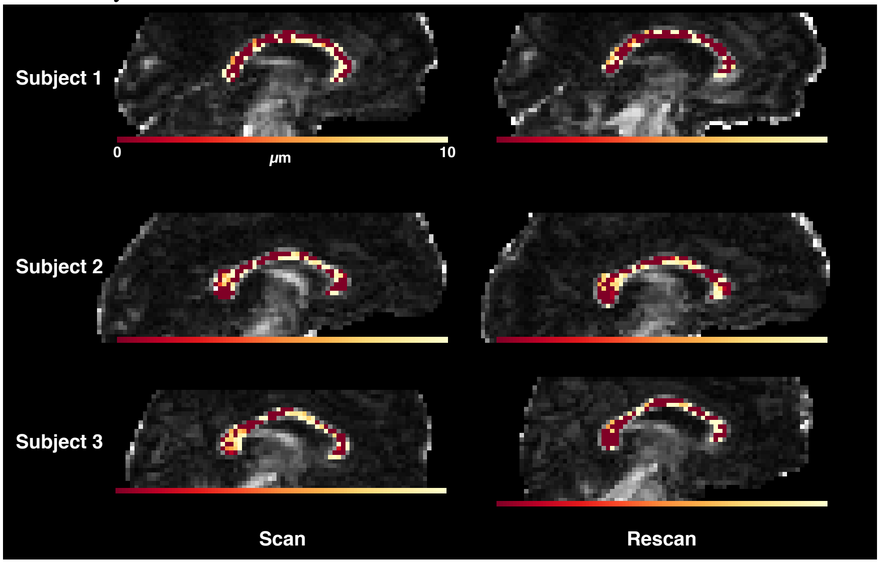

Supplement: Supplementary file 1 — Figure S1: Maps of axon radius index, estimated in the absence of the prior on R. Compared to Fig. 5, the maps are noisier and show less scan–rescan consistency. [file MRM-75-1787-s001.pdf]
